# Effects of prophylactic and therapeutic antimicrobial uses in small‐scale chicken flocks

**DOI:** 10.1111/zph.12839

**Published:** 2021-05-02

**Authors:** Nguyen Van Cuong, Bach Tuan Kiet, Doan Hoang Phu, Nguyen Thi Bich Van, Vo Be Hien, Guy Thwaites, Juan Carrique‐Mas, Marc Choisy

**Affiliations:** ^1^ Oxford University Clinical Research Unit Ho Chi Minh City Vietnam; ^2^ Sub‐Department of Animal Health and Production (SDAHP) Cao Lanh Vietnam; ^3^ Faculty of Animal Science and Veterinary Medicine University of Agriculture and Forestry Ho Chi Minh City Vietnam; ^4^ Centre for Tropical Medicine and Global Health Nuffield Department of Medicine University of Oxford Oxford UK

**Keywords:** AMU, chicken, prophylactic, therapeutic, Vietnam

## Abstract

Antimicrobials are extensively used both prophylactically and therapeutically in poultry production. Despite this, there are little data on the effect of antimicrobial use (AMU) on disease incidence rate and per cent mortality. We investigated the relationships between AMU and disease and between AMU and mortality using data from a large (*n* = 322 flocks) cohort of small‐scale chicken flocks in the Mekong Delta, Vietnam, that were followed longitudinally from day old to slaughter (5,566 observation weeks). We developed a parameterized algorithm to emulate a randomized control trial from observational data by categorizing the observation weeks into ‘non‐AMU’, ‘prophylactic AMU’ and ‘therapeutic AMU’. To evaluate the prophylactic AMU effect, we compared the frequencies of clinical signs in ‘non‐AMU’ and ‘prophylactic AMU’ periods. To analyse therapeutic AMU, we compared weekly per cent mortality between the weeks of disease episodes before and after AMU. Analyses were stratified by clinical signs (4) and antimicrobial classes (13). Prophylactic AMU never reduced the probability of disease, and some antimicrobial classes such as lincosamides, amphenicols and penicillins increased the risk. The risk of diarrhoea consistently increased with prophylactic AMU. Therapeutic AMU often had an effect on mortality, but the pattern was inconsistent across the combinations of antimicrobial classes and clinical signs with 14/29 decreasing and 11/29 increasing the per cent weekly mortality. Lincosamides, methenamines and cephalosporins were the only three antimicrobial classes that always decreased the mortality when used therapeutically. Results were robust respective to the parameters values of the weeks categorization algorithm. This information should help support policy efforts and interventions aiming at reducing AMU in animal production.

**Impact**

- This study uses a large volume of observational data on disease and antimicrobial usage in small‐scale chicken farms of the Mekong Delta region of Vietnam in order to quantify the prophylactic and therapeutic effects of antimicrobials from 13 different classes on diarrhoea, respiratory infections, legs lesions and central nervous system infections.
- We show that prophylactic antimicrobial use never reduced the risk of diseases and that some classes actually increased the risk of some diseases (e.g. diarrhoea).
- In small‐scale flock settings, the therapeutic use of antimicrobials leads to an increase in mortality in about 50% of the investigated antimicrobial/disease combinations.

## 1 INTRODUCTION

Antimicrobials play a critical role in the maintenance of animal health, animal welfare and food safety (FAO, 2016) and are used worldwide in food‐producing animals for the prevention and treatment of infectious diseases. In addition, in some countries, they are also added to commercial feed rations as antimicrobial growth promoters (AGPs) (Landers et al., 2012). Consumption of antimicrobials in animal production has been predicted to increase by two thirds from 2010 to 2030, of which one third is likely to include antimicrobial usage (AMU) for disease prevention and growth promotion purposes (or sub‐therapeutic doses), especially in pig and poultry production (Van Boeckel et al., 2015). These predictions are, however, uncertain, given that many countries have started to implement restrictions especially with regard to AGPs.

In veterinary medicine, non‐therapeutic administration of antimicrobials to individual animals is common in companion, bovine and equine medicine to prevent surgical site infections (Duclos et al., 2017; Dumas et al., 2016). In food animals, antimicrobials are often used to prevent bacterial infections (prophylactically) and also after potential exposure to a pathogen to reduce clinical signs and/or mortality (metaphylactically) (Pagel & Gautier, 2012; Rerat et al., 2012). Regardless of its purpose, in our study farms, antimicrobials are typically administered to whole flocks via drinking water, making it difficult to distinguish therapeutic from metaphylactic use at flock level and both are generally indistinctly called therapeutic. Thus, in the rest of this article, we define prophylactic or therapeutic use in relation to the use of antimicrobials before or after the onset of disease (i.e. clinical signs). Prophylactic AMU in poultry flocks often takes place during the brooding period and during other key events of the flocks’ life such as vaccination and prior to transport. In a recent study of 203 small‐scale commercial flocks (of 102 farms) in the Mekong Delta region of Vietnam, antimicrobials were extensively used and the highest frequency of AMU corresponded to the brooding period.

The practice of prophylactic medication of flocks/herds is likely to promote a shift in enteric bacterial populations from susceptible towards resistance. This is likely to have potential public health implications (Lugsomya et al., 2018).

There are limited data on the identity of pathogens circulating in the area. A study identified a range of global pathogens in diseased flocks in the study area, the most common being, in descending order, *Avibacterium* *paragallinarum* (62.3% flocks), followed by *Mycoplasma* *gallisepticum* (26.2%), infectious bursal disease (24.6%) and infectious bronchitis (21.3%). However, the diagnostic panel was limited to nine pathogens and it is likely that many more pathogens are circulating in the area. Furthermore, the pathogens are likely to change over time. In 47.5% of disease episodes, more than one aetiological cause was found (BichVan et al., 2019). However, the exact reason for AMU (i.e. prophylactic versus therapeutic) in flocks remains unclear (Carrique‐Mas et al., 2015; Cuong et al., 2019). Despite extensive use of antimicrobials in poultry production, there are little empirical data on the overall effects of prophylactic and therapeutic AMU on flock health. A recent study in Dutch layer chicks indicated that early mass prophylactic antimicrobial administration had a negative impact on adaptive immunity later in life (Simon et al., 2016).

Here, we analysed observational data on AMU and disease (clinical signs) collected from a large cohort of small‐scale chicken commercial flocks in the Mekong Delta of Vietnam (Cuong et al., 2019). We aimed to estimate (a) the effect of prophylactic AMU on the subsequent probability of occurrence of a disease episode and (b) the impact of therapeutic AMU on subsequent mortality rate during a disease episode. In order to make causal inference from observational data, we developed a parameterized algorithm that emulates a randomized control trial from these observational data, as proposed by Glass et al. (2013). We also explored the robustness of our results respective to the exact values of the parameters of our algorithm. The analyses were stratified by classes of antimicrobial active ingredient (AAI) and specific type of clinical sign. These results provide a scientific basis that underpins policies aimed at reducing prophylactic AMU in farming systems.

## 2 MATERIAL AND METHODS

### 2.1 Data collection

Data on AMU, disease (clinical signs) and mortality from a random selection of commercial small‐scale native chicken flocks raised for meat in Dong Thap province (Mekong Delta of Vietnam) were used. Farmers listed in the official census were initially contacted and invited to join the study. The data collection methods have been described elsewhere (Cuong et al., 2019). In brief, farmers were provided with a structured diary and were trained by project veterinarians to identify and record the most common clinical signs of disease, as well as to weekly record information on AMU and number of dead animals. The clinical signs recorded were (a) respiratory distress (sneezing, coughing, nasal/ocular discharge, difficult breathing), (b) diarrhoea (watery faeces), (c) alterations of the central nervous system (CNS) (ataxia, circling, torticollis) and (d) leg lesions (lameness, swollen joints/foot pads). Antimicrobial active ingredients (AAIs) were grouped by antimicrobial classes based on World Organization for Animal Health (OIE) criteria (OIE, 2015). A total of 5,566 weeks of data were collected from 322 flock cycles raised in 116 farms. The data were collected from October 2016 until May 2019. This is an observational study and thus did not require institutional review board approval.

### 2.2 Analyses

The main challenge of the analysis consists in emulating a randomized controlled trial from our observational data. Below we explain in detail how this is performed.

The statistical unit in this study is a week of observation. The main challenge of the analyses is that antimicrobials were administered without mentioning the purpose of use (prophylactic or therapeutic). We thus had to use the information on the timing of presence of disease and AMU in order to categorize each week of the data set into three categories: ‘non‐AMU’ (used as control), ‘prophylactic AMU’ and ‘therapeutic AMU’. Note that not all weeks could be assigned to one of these three categories as explained in the paragraph below that describes in detail the categorization algorithm.

**FIGURE 1:**
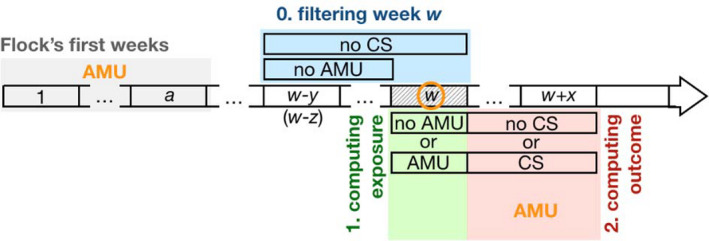
Data preparation for the estimation of the prophylactic effect of AMU. The horizontal arrow represents the time line of a flock, divided into weeks, represented by rectangles, starting on week 1 (on the left). For any given week *w* selected (by step 0, see below) for the analysis (represented here by the hashed rectangle), we computed (i) an exposure variable based on the use or not of antimicrobials (step 1, in green) and (ii) an outcome variable based on the occurrence or not of clinical signs over an observation period of *x* weeks after week *w* (step 2, in red). Statistical analyses then tested whether AMU on week *w* (exposure) affects the occurrence of clinical signs over the observation period (outcome). In order to make sure that AMU exposure on week *w* does correspond to prophylactic AMU, we filtered out all the weeks that were preceded by (i) the presence of clinical signs over a period of *y* weeks before week *w* (including week *w*) or (ii) AMU over a period of *z* weeks before week *w* (naturally excluding the candidate week, since this information is used to compute the exposure variable). This step 0 is shown in blue on the figure. Finally, the analysis includes potential confounding factors (shown in orange letters and circle) such as the age of the chicken (i.e. week *w*) as well as AMU during the first *a* weeks of the flock's life (brooding period, in grey) and during the *x* weeks of the observation period

For the prophylactic AMU analysis, we considered only weeks (a) without clinical signs reported during that week, as well as during the *y* preceding weeks, and (b) without any antimicrobials being used during the *z* preceding weeks (filtering, step 0 on Figure 1). These selected weeks were then labelled as ‘with AMU’ or ‘without AMU’, depending on whether they had or not had AMU (exposure, step 1 on Figure 1) and, for each of these weeks, we computed the occurrence of clinical signs during the *x* subsequent weeks of observation (outcome, step 2 on Figure 1). The analyses were additionally adjusted for three covariables in order to control for potential confounders: (a) AMU during the first *a* weeks of the flock (brooding period), (b) AMU during the *x* weeks of the observation period and (c) flock age (all in orange on Figure 1). Comparisons were performed by building a logistic generalized additive model with the probability of a disease episode as the dependent variable and in which the potential non‐linear effect of age was modelled using a spline‐based smoothing function, the optimal degree of which was obtained by cross‐validation as implemented by the mgcv R package (Wood, 2017).

**FIGURE 2:**
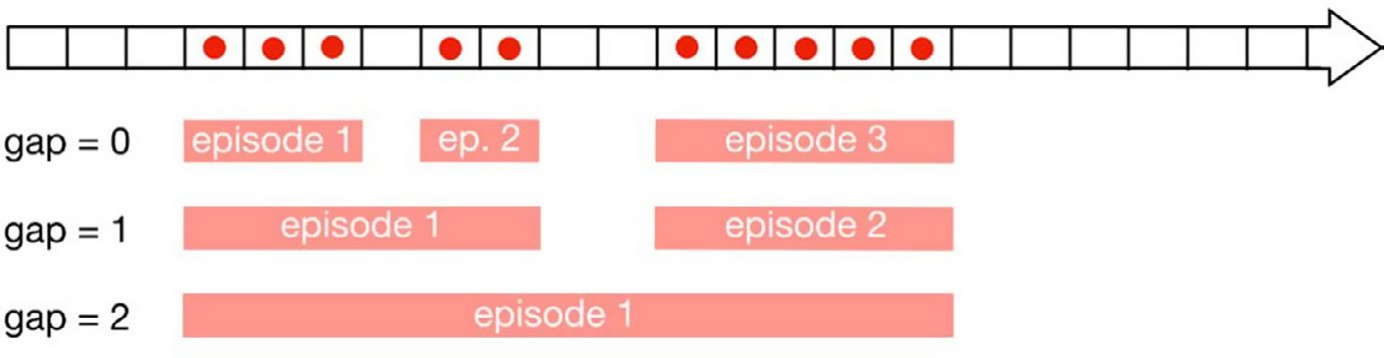
Defining a disease episode. The horizontal arrow represents the time line of a flock, divided into weeks, represented by rectangles, from the first week on the left to the last one on the right. The red dots represent the reporting of disease (clinical sign). In order to account for the fact that clinical signs may not be always reported, we allow the possibility to convert one or a few consecutive weeks without reported clinical signs and surrounded by weeks with reported clinical signs into one single disease episode. The gap parameter is the number of consecutive week(s) without clinical signs we allow when defining a disease episode. Below the time line arrow are three examples of disease episodes definitions: three episodes when maximum gap = 0 (top), two episodes when maximum gap = 1 (middle) and one episode only when maximum gap = 2 (bottom)

**FIGURE 3:**
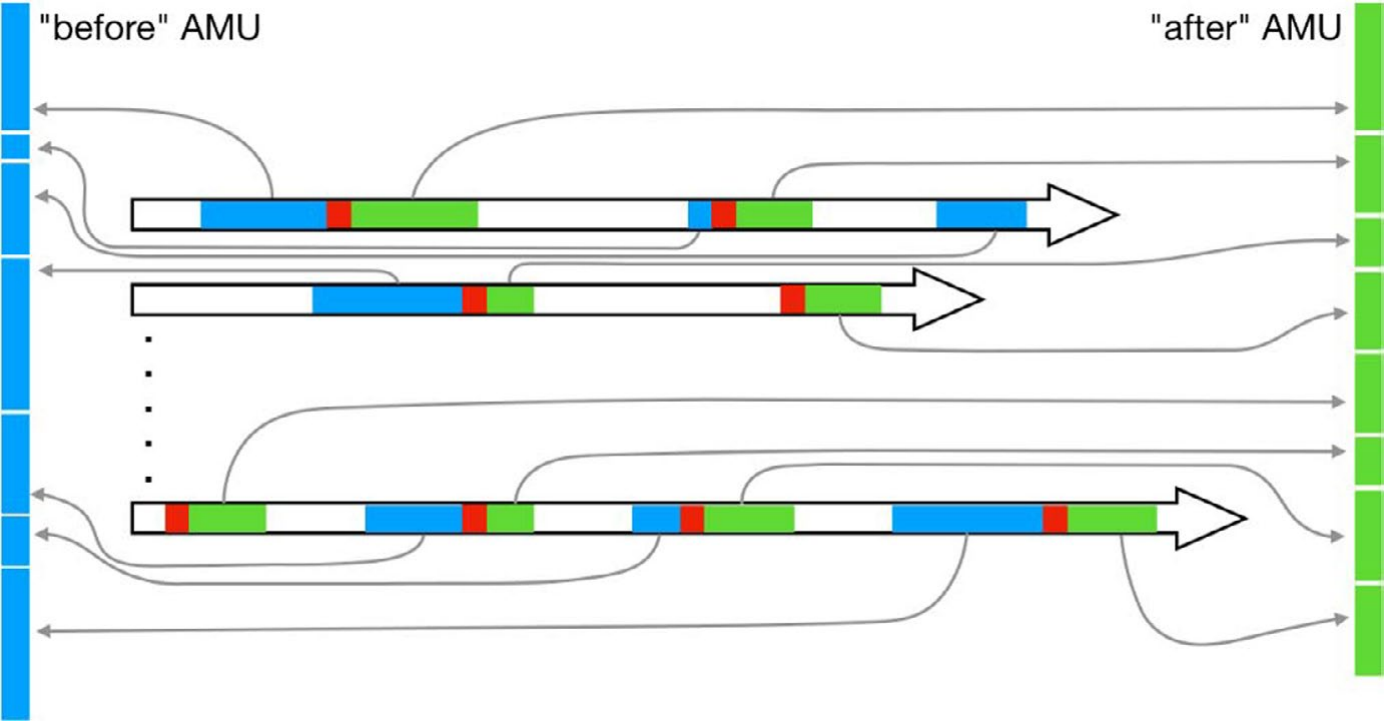
Separating ‘before AMU’ and ‘after AMU’ arms in all the disease episodes. In this example, the horizontal arrows show the first 2 (top) and the last (bottom) flocks of the data set. Each flock starts on the left end and ends on the right end of the arrow, and the length of the arrow is the duration of the flock. Coloured sections represent disease episodes as identified on Figure 2. The red rectangles represent the first week of AMU (if any) in the disease episodes. Sometimes, there is no AMU at all during the disease episode (as on the third episode of the first flock), and some other times, the first week of AMU is the first week of the episode (as on the second episode of the second flock or the first episode of the last flock). Once these first weeks of AMU are identified in all the disease episodes, we gathered, from all the disease episodes of all the flocks, all the weeks that occur before (in blue) in one arm ‘before’, and all the weeks that occur after (in green) these first weeks of AMU in another arm ‘after’

For the analysis of therapeutic AMU (i.e. therapeutic and metaphylactic combined), the statistical units were the weeks of an episode of disease, defined as a series of consecutive weeks with clinical signs recorded in a flock. Because clinical signs are likely to be under‐reported, we allowed for the possibility of presence of weeks without any disease reporting in the middle of disease episodes. Figure 2 shows three examples of definition of disease episodes allowing gaps of 0, 1 and 2 consecutive weeks without any disease report. The weeks of disease episodes were then grouped into two arms (exposure): one with all the weeks (in blue on Figure 3) before the onset of AMU (if any, in red on Figure 3) in the disease episode and the other one with all the weeks (in green on Figure 3) following onset of AMU (if any, in red on Figure 3). In case of absence of AMU during the disease episode, all the weeks were assigned to the first arm. In order to ensure that AMU can be considered as therapeutic, we excluded from the analysis all the weeks where other antimicrobials were used during the *p* weeks that preceded. The per cent weekly mortality (proportion of chickens dying each week) was computed for the two arms and was compared using a logistic generalized additive model that included the spline‐based smoothed age of the flock as a covariable as described above for the characterization of the prophylactic effect of AMU.

The two analyses included a number of tuning parameters. For the prophylactic AMU analysis, these were *x*, the duration (in weeks) of the observation period; *y* and *z*, the numbers of weeks filtering for previous presence of clinical signs and AMU, respectively; and *a*, the duration of the first few weeks of the flock during which we look for potential AMU. For the therapeutic analysis, we set a gap *g* (in weeks) to define disease episodes and *p*, the number of weeks filtering for previous AMU. Furthermore, in both analyses, disease is defined by the presence of at least one of a set of clinical signs, and AMU is defined by the use of at least one of a set of antimicrobials. In absence of information on what the values of these tuning parameters should be, we considered various combinations of them in order to assess the robustness of our results. For the prophylactic AMU analysis, we considered all the combinations (*n* = 27) of *x* = 1, 2, 3, *y* = *z* = 1, 2, 3, and *a* = 1, 2, 3. For the therapeutic analysis, we considered all the combinations (*n* = 9) of *g* = 0, 1, 2 and *p* = 1, 2, 3. We performed the analyses separately for each antimicrobial class (*n* = 13) and type of clinical sign (*n* = 4), as well for any AMU and any clinical signs.

## 3 RESULTS

### 3.1 Data on AMU and clinical signs

Antimicrobials were administered to a total of 296/322 (91.9%) flocks and on 1,266/5,566 (22.7%) observation weeks. A total of 44 different AAIs corresponding to 13 antimicrobial classes were used, with tetracyclines, polypeptides, aminoglycosides, macrolides and penicillins being the most commonly used classes (both by flock and by week, Table 1). In addition, clinical signs were reported on 530/5,566 (9.5%) weeks, with diarrhoea on 305 (5.5%) weeks, respiratory on 213 (3.8%) weeks, leg lesions on 71 (1.3%) weeks and CNS on 51 (0.9%) weeks.

**TABLE 1:** Description of the AMU data, including data for prophylactic AMU analysis (No. weeks with prophylactic AMU and number of weeks without AMU) and therapeutic AMU analysis (No. weeks before and after therapeutic AMU). The data are stratified by antimicrobial class (by row). The ranges reflect the variability resulting from different combinations of the tuning parameters of the categorization algorithm

Classes	raw AMU data		Data for prophylactic effect analysis		Data for therapeutic effect analysis		
	No. flocks *N* = 349 (%)	No. weeks *N* = 5,566 (%)	No. weeks with prophylactic AMU (%)	No. weeks with non‐AMU (%)	No. disease episodes	No. weeks before therapeutic AMU	No. weeks after therapeutic AMU
Aminoglycosides	158 (45.3)	367 (6.6)	125–250 (34.1–68.1)	2,841–4,554 (54.6–87.6)	44–100	53–396	0–103
Amphenicols	66 (18.9)	100 (1.8)	51–80 (51–80)	3,236–4,984 (59.2–91.2)	13–30	44–541	0–35
Cephalosporins	7 (2.0)	9 (0.2)	2–8 (22.2–88.9)	3,412–5,139 (61.4–92.5)	0–1	298–623	0–4
Diaminopyrimidines	56 (16.0)	106 (1.9)	33–74 (31.1–69.8)	3,258–4,978 (59.7–91.2)	18–34	191–568	0–29
Lincosamides	24 (6.9)	33 (0.6)	15–30 (45.5–90.9)	3,367–5,093 (60.9–92)	4–9	197–607	0–9
Macrolides	137 (39.3)	310 (5.6)	117–220 (37.7–71)	2,909–4,638 (55.3–88.2)	22–68	45–478	2–53
Penicillins	113 (32.4)	208 (3.7)	95–160 (45.7–76.9)	3,023–4,791 (56.4–89.4)	34–61	145–498	0–57
Pleuromutilins	1 (0.3)	1 (0.0)	1–1 (100–100)	3,419–5,154 (61.4–92.6)	0–0	0–0	0–0
Polypeptides	252 (72.2)	605 (10.9)	261–407 (43.1–67.3)	2,249–4,158 (45.3–83.8)	63–133	37–340	0–109
Quinolones	98 (28.1)	168 (3.0)	74–129 (44–76.8)	3,113–4,870 (57.7–90.2)	20–39	42–538	2–50
Sulphonamides	83 (23.8)	148 (2.7)	65–109 (43.9–73.6)	3,138–4,906 (57.9–90.6)	22–50	46–527	1–52
Tetracyclines	258 (73.9)	628 (11.3)	266–432 (42.4–68.8)	2,223–4,117 (45–83.4)	57–127	43–339	0–136
Methenamines	26 (7.4)	36 (0.6)	15–31 (41.7–86.1)	3,356–5,091 (60.7–92.1)	7–16	201–619	0–1
Any class	296 (84.8)	1,266 (22.7)	353–686 (27.9–54.2)	1,564–3,251 (36.4–75.6)	144–310	21–164	1–153

Note

Antimicrobial agents within each class: Aminoglycosides: neomycin, gentamicin, streptomycin, spectinomycin, apramycin, josamycin. Amphenicols: florfenicol, thiamphenicol, chloramphenicol. Cephalosporins: cefadroxil, cefotaxime, cephalexin, ceftiofur. Diaminopyrimidines: trimethoprim. Lincosamides: lincomycin. Macrolides: tylosin, tilmicosin, erythromycin, spiramycin, kitasamycin. Penicillins: amoxicillin, ampicillin. Pleuromutilins: tiamulin. Polypeptides: colistin, enramycin. Quinolones: enrofloxacin, flumequine, norfloxacin. Sulphonamides: sulphachloropyridazine, sulphadiazine, sulphadimethoxine, sulphadimidine, sulphaguanidine, sulphamethazine, sulphamethoxazole, sulphamethoxypyridazine, sulphamethoxazole, sulphathiazole. Tetracyclines: oxytetracycline, doxycycline, tetracycline. Methenamines: methenamine.

### 3.2 Data for prophylactic and therapeutic AMU analysis

Depending on the values of the tuning parameters, 353–686 (27.9%–54.2%) of the 1,266 AMU weeks were classified as prophylactic AMU. The highest frequency of prophylactic AMU corresponded to tetracyclines, polypeptides, aminoglycosides and macrolides classes. A range of 1,564–3,251 (36.4%–75.6%) of all the 5,566 weeks was classified as non‐AMU. A range of 144–310 disease episodes was identified. The highest frequencies of first week therapeutic AMU corresponded to tetracyclines, polypeptides, aminoglycosides and penicillins class. Ranges of 21–164 and 1–153 weeks were classified as weeks ‘before’ and ‘after’ therapeutic AMU, respectively. The details of the data used for each class of antimicrobial are presented in Table 1.

### 3.3 Impact of prophylactic AMU on disease occurrence

**FIGURE 4:**
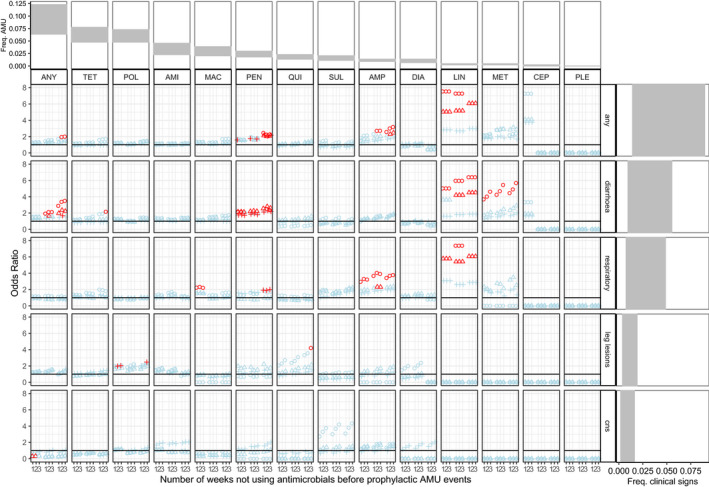
OR (Odds Ratios) of occurrence of clinical signs when antimicrobials were used prophylactically. For the ease of visualization, confidence intervals are not represented. Instead, red colour indicates OR values that are statistically significant (*p* < .05) and light blue colour indicates OR values that are not significant. Circle, triangle and cross shapes represent durations *x* of the observation period equal to 1, 2 and 3 weeks, respectively. Numbers 1, 2 and 3 represent the duration *y* = *z* of the filtering period (the number of weeks without any AMU before prophylactic events). In each subpanel, each combination of three numbers 123 represented, from left to right, the AMU in the first 1, 2 and 3 weeks of life. The horizontal black line represents an OR value of 1. OR values higher than the horizontal black line indicate that the prophylactic AMU increases the risk of having clinical signs. A linear scale instead of a logarithm one was chosen for the OR in order to show the spread of significant values better. Antimicrobial classes were ordered from the most to the least commonly used. Abbreviations: ‘ANY’ = any classes, ‘AMI’ = aminoglycosides, ‘AMP’ = amphenicols, ‘CEP’ = cephalosporins, ‘DIA’ = diaminopyrimidines, ‘MAC’ = macrolides, ‘MET’ = methenamines, ‘LIN’ = lincosamides, ‘PLE’ = pleuromutilins, ‘POL’ = polypeptides, ‘QUI’ = quinolones, ‘SUL’ = sulphonamides, ‘TET’ = tetracyclines, ‘Freq. AMU’ = Frequency AMU, ‘Freq. clinical signs’ = Frequency clinical signs. First row and right column, respectively, show the ranges of frequencies of AMU and clinical signs observed in the study farms

Figure 4 shows the odds ratio (OR) of the effects of prophylactic AMU per antimicrobial class and clinical sign, and for all the combinations of the tuning parameters. None of the prophylactic AMU ever protects (i.e. OR significantly below 1) from any of the clinical signs. On the contrary, in 10 of the 52 antimicrobial class x clinical sign combinations, prophylactic AMU actually increases the probability of occurrence of disease. Only the CNS was never affected. The risk of diarrhoea increased with the prophylactic use of lincosamides, methenamines and pleuromutilins. The risk of respiratory infections increased with the prophylactic use of lincosamides and amphenicols. The significances of these effects are higher for short observation periods and longer initial period of flocks. The duration of the filtering period has little effect of the significance of these results.

### 3.4 Impact of therapeutic AMU on mortality

**FIGURE 5:**
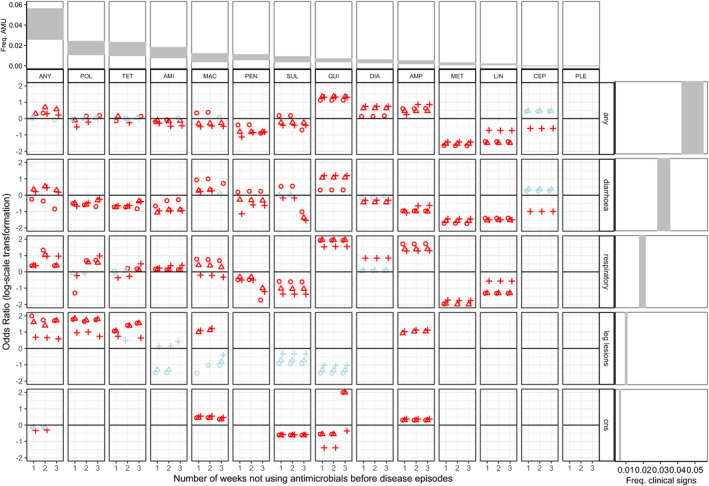
OR (Odds Ratio) of the impact of therapeutic AMU on mortality. For the ease of visualization, confidence intervals are not represented. Instead, red colour indicates OR values that are statistically significant (*p* < .05) and light blue colour indicates OR values that are not significant. Cross, circle and triangle shape represented 0, 1 and 2 weeks of gap in disease episodes, respectively. The horizontal black line represents an OR of 1. OR values higher than the horizontal black line indicate that therapeutic AMU increases the mortality rate. Antimicrobial classes were ordered from the most to the least commonly used. Abbreviations: ‘ANY’ = any classes, ‘AMI’ = aminoglycosides, ‘AMP’ = amphenicols, ‘CEP’ = cephalosporins, ‘DIA’ = diaminopyrimidines, ‘MAC’ = macrolides, ‘MET’ = methenamines, ‘LIN’ = lincosamides, ‘PLE’ = pleuromutilins, ‘POL’ = polypeptides, ‘QUI’ = quinolones, ‘SUL’ = sulphonamides, ‘TET’ = tetracyclines. First row and right column, respectively, show the ranges of frequencies of AMU and clinical signs observed in the study farms

Figure 5 shows the odds ratio of the effects of therapeutic AMU on per cent weekly mortality, stratified by antimicrobial class and clinical sign, and for all the combinations of the tuning parameters. Therapeutic AMU almost always has an effect on the mortality rate. However, this effect varies both between and within antimicrobial classes and clinical signs combinations. Out of the 31 combinations for which we have data, only two do not show any significant results. Among the 29 other ones, 11 showed robust increase in mortality rate (respective to the exact values of the tuning parameters), 14 showed robust decrease in mortality rate, and four showed inconsistent results depending of the values of the tuning parameters. The effects of the tuning parameters on the significance of the results were not consistent from combination to combination of antimicrobial classes and clinical signs. Lincosamides and methenamines always decrease the mortality, and this is fairly robust respective to the exact values of the tuning parameters. AMU in response to leg lesions always increases mortality.

## 4 DISCUSSION

Based on disease reporting data collected longitudinally from chicken flocks, our study suggests that prophylactic AMU does not protect against disease. Instead, we found that prophylactic AMU did increase the risk of disease in a number of situations. Specifically, we found that some of the antimicrobial classes administered prophylactically resulted in increased risk of subsequent diarrhoea (lincosamides, penicillins, methenamines and tetracyclines classes) and respiratory infections (lincosamides, penicillins, amphenicols and macrolides). The association between AMU and diarrhoea has a biological basis, since microbial communities of the gastro‐intestinal tract of chickens play an important role in nutrient digestion, pathogen inhibition and interact with the gut‐associated immune system (Borda‐Molina et al., 2018). These results are also consistent with previous studies: oral administration of clindamycin (lincosamide class) in humans results in considerable alterations of the intestinal microbiota even long after discontinuation of the antimicrobial course (Jakobsson et al., 2010). A study on pigeons receiving this drug resulted in an increased risk of secondary yeast infection, resulting in diarrhoea and sour crop (Lenarduzzi et al., 2011). Similarly, the therapeutic use of methenamines, tetracyclines and broad‐spectrum penicillins in humans has been shown to have enteric side effects (Chwa et al., 2019; Rafii et al., 2008).

Our analyses also show that the significance of the effect of prophylactic AMU on clinical signs tends to decrease as the duration of the observation period increases, suggesting that the effect of AMU may be of relatively short term, typically 2 weeks. It is believed that antimicrobials may trigger dysbiosis, which may impact host systemic energy metabolism and cause phenotypic and health modifications (Le Roy et al., 2019). Furthermore, a study indicated that bacterial phylotypes shifted after 14 days of antimicrobial treatment in pigs (Looft et al., 2012) and 7 days in humans (Jakobsson et al., 2010).

The effect of prophylactic AMU on clinical signs increased with the duration of the brooding period. AMU during the first weeks of life has been reported to decrease the diversity of intestinal microbiota, which may have health consequences later in life (Kers et al., 2018). An additional explanation would be the potential of antimicrobials to reduce the immune response of chickens. A study in broiler flocks showed that haematological values fell after the administration of antimicrobials to young chicks (1–5 days old) (Al‐Saad & A.A. Yones, 2014).

Contrary to the effect of prophylactic AMU on disease occurrence, the effect of therapeutic AMU on mortality was almost always significant. However, the general picture was less clear‐cut than for prophylactic effects as it varied greatly both within and between combinations of antimicrobial classes and clinical signs, as well as depending on the values of the tuning parameters. Therapeutic AMU always resulted in increased mortality among flocks affected by leg lesions. For the three other clinical signs, it depended on the antimicrobial class. Lincosamides and methenamines always decreased mortality. The effects of the other antimicrobial classes depended on the clinical signs under consideration. Interestingly, lincosamides and methenamines were also two classes that conferred the highest risk of subsequent disease when used prophylactically. In our study farms, these two classes had a comparatively low level of usage both in terms of frequency and amount (Cuong et al., 2019). We speculate that low levels of usage may have selected little resistance in the microbiota of our study flocks, resulting in a comparatively more potent effect associated with these two antimicrobial classes. Demonstrating this, however, is not easy given the vast range of potential pathogens and commensal organisms colonizing chicken flocks.

In addition to bacterial infections, other possible causes of diarrhoea in poultry include coccidiosis, helminths and viruses (such as rotavirus and adenovirus). Antimicrobials will not eliminate these non‐bacterial pathogens but might help to prevent superinfections. Indeed, the pathogens listed above tend to damage the chicken intestine which allows harmful bacteria to grow out of control in the intestine, leading to diarrhoea, increasing the disease severity and ultimately the risk of death.

Leg problems may be caused by a range of aetiologies including bacterial, viral diseases as well as metabolic and nutritional disorders. The observed association between AMU and leg disease is consistent the involvement of non‐bacterial pathogens such as Marek virus (leg paresis) and reovirus (viral arthritis with severe lameness and swollen hock) or metabolic/nutritional disorders in the aetiology of these problems.

For episodes of respiratory and CNS diseases, there was not a clear association between therapeutic AMU and mortality, suggesting that, in our setting, primarily non‐bacterial pathogens may be responsible for such infections (i.e. avian influenza, Newcastle, infectious bronchitis, infectious laryngotracheitis, fowlpox, etc.). In the case of respiratory diseases, complex bacterial–viral–vaccine interactions and common, and therefore, AMU may not contribute to mitigate the mortality outcome. A recent study has demonstrated the diverse number of viral pathogens that typically affect chickens with respiratory disease in the area (BichVan et al., 2019; Choisy et al., 2019).

To our knowledge, this is the first epidemiological study addressing the impact of prophylactic and therapeutic AMU on the health status of chicken flocks from a low‐ and middle‐income country. The approach we used to define prophylactic and therapeutic AMU (with a pre‐selection of weeks) was possible because of the high volume of data collected on a weekly basis (>5,000weeks). A structural limitation of the data is that when both AMU and clinical signs were reported on the same week for the first time in a flock, it was not possible to determine which of the two events occurred first. Because of this, about 50% of the data were excluded, thus decreasing the statistical power of the study. In addition, in most cases, antimicrobial products included two or more AAIs, and disease episodes presented with a combination of different clinical signs. Given the large number of combinations possible, we restricted our analyses to examining the impact of AMU by class on individual clinical signs. Our study also excluded any antimicrobials present in feed as AGPs. This is the case for about 40% of the feed formulatins examined. However, the concentrations (strength) of antimicrobials included in these feeds are much lower than the ones used prophylactically. The reason why we did not attempt to measure AGP consumption was that farms often use different feed formulations simultaneously and the labelling is ambiguous (e.g. this feed product may include one of the following antimicrobials: A, B or C). As many other countries worldwide, Vietnam is also currently engaged in legislative efforts leading to progressive reductions of antimicrobials. In Vietnam, a recent decree (13/2020/ND‐CP) includes the time frame for a ban of AMU for prophylactic purposes (including AGPs), with phased bans for different antimicrobials classes: WHO ‘highest’ and ‘high priority’ critically important AAIs to be banned from 2021, highly important AAIs from 2022, important AAIs from 2023 and all other antimicrobial classes from of 2026. We do not know, however, the level of compliance with this upcoming legislation.

## 5 CONCLUSIONS

We found evidence that prophylactic AMU does not prevent infection and may instead increase the risk of clinical disease in chicken flocks. In general, prophylactic use of lincosamides, penicillins, methenamines and tetracyclines increased the risk of diarrhoea, and prophylactic use of lincosamides, penicillins, macrolides and amphenicols increased the risk of respiratory infections. Therapeutic use of any antimicrobial class resulted in an overall increase in mortality. The majority of antimicrobial classes reduced mortality associated with diarrhoeal infections. However, therapeutic use of antibiotic in response to leg problems resulted in increased risk of death. For respiratory and CNS infection, the outcome of therapeutic AMU was inconsistent and unpredictable, even within a single class of antimicrobials. Lincosamides, methenamines and cephalosporins were the only antimicrobial classes that always decreased mortality when used therapeutically. Lincosamides and methenamines were also the two classes of antimicrobials that increased the risk of disease the most when used prophylactically. These results should support the principle that farmers should in general avoid prophylactic use of antimicrobials and focus instead on improving husbandry practices.

## CONFLICT OF INTEREST

The authors declare that they have no conflicts of interest in relation to this paper.

## Data Availability

The code and data used for this article are freely available from http://doi.org/10.5281/zenodo.4638391
